# Mobile Apps for Health Behavior Change: Protocol for a Systematic Review

**DOI:** 10.2196/16931

**Published:** 2020-01-30

**Authors:** Madison Milne-Ives, Ching Lam, Michelle Helena Van Velthoven, Edward Meinert

**Affiliations:** 1 Department of Paediatrics Digitally Enabled Preventative Health Research Group University of Oxford Oxford United Kingdom; 2 Department of Primary Care and Public Health Imperial College London London United Kingdom

**Keywords:** telemedicine, mobile health, mobile apps, cell phone, health behavior, intervention

## Abstract

**Background:**

The popularity and ubiquity of mobile apps have rapidly expanded in the past decade. With a growing focus on patient interaction with health management, mobile apps are increasingly used to monitor health and deliver behavioral interventions. The considerable variation in these mobile health apps, from their target patient group to their health behavior, and their behavioral change strategy, has resulted in a large but incohesive body of literature.

**Objective:**

The purpose of this protocol is to provide an overview of the current landscape, theories behind, and effectiveness of mobile apps for health behavior change.

**Methods:**

The Preferred Reporting Items for Systematic Reviews and Meta-Analyses Protocols will be used to structure this protocol. The focus of the systematic review is guided by a population, intervention, comparator, and outcome framework. A systematic search of Medline, EMBASE, CINAHL, and Web of Science will be conducted. Two authors will independently screen the titles and abstracts of identified references and select studies according to the eligibility criteria. Any discrepancies will then be discussed and resolved. One reviewer will extract data into a standardized form, which will be validated by a second reviewer. Risk of bias was assessed using the Cochrane Collaboration Risk of Bias tool, and a descriptive analysis will summarize the effectiveness of all the apps.

**Results:**

As of November 2019, the systematic review has been completed and is in peer review for publication.

**Conclusions:**

This systematic review will summarize the current mobile app technologies and their effectiveness, usability, and coherence with behavior change theory. It will identify areas of improvement (where there is no evidence of efficacy) and help inform the development of more useful and engaging mobile health apps.

**Trial Registration:**

PROSPERO CRD42019155604; https://tinyurl.com/sno4lcu

**International Registered Report Identifier (IRRID):**

PRR1-10.2196/16931

## Introduction

Health care is becoming increasingly more digital. Digital interventions to engage patients with their health management have become popular, particularly since the first smartphone (iPhone) was released in 2008. Patient engagement has become a focus of health care research and has a significant role in preventative health care [1,2]. Recently, there has been much research about the use of digital technologies to enhance specific health behaviors [3,4]. Positive health behaviors include a wide variety of actions aimed at preventing problems and maintaining and improving patients’ health [5]. This covers a range of lifestyle choices and health condition management: primarily drug and alcohol use, diet, and physical activity, although other behaviors such as sun protection, sex safety, medication adherence, doctor’s appointments, vaccinations, self-management of chronic conditions, and mental health can also be included [6,7].

Thousands of different internet- and mobile-based interventions, including apps, texting, emails, social media platforms, voice agents, and customized interactive chatbots, have been developed to help patients improve their health behaviors [8-11]. Given the widespread ownership, frequent use, and computing power of smartphones and a large number of currently available mobile health apps [12], they are a particularly promising platform for targeted interventions. Smartphones can be used to collect data, including tracking physical activity, mental health, sleep patterns, and basic physiological measures, and make inferences about health and well-being [13]. Apps can also connect patients with their health information and health care providers, primarily by allowing them to access and contribute to their electronic health records [13,14]. However, mobile health apps are most commonly used to deliver interventions aimed at improving a wide variety of patients’ health behaviors [15]. Concerningly, many of the current interventions are not based on any behavior change theories or techniques, and their effectiveness is not correctly evaluated [9,14,16]. Thus, there is a great scope for mobile health (mHealth) apps to improve their abilities to promote and maintain positive health behavior change.

A search of “mobile health” or “mHealth” on PROSPERO reveals many systematic reviews that are currently exploring mobile health interventions. However, with few exceptions [14,17], these systematic reviews have a narrow focus on particular types of health behaviors, patient groups, or a combination of both. Han and Lee conducted a systematic review in 2017 to examine the effectiveness of mobile health apps to improve patients’ health behaviors [14]. The authors only included randomized controlled trials (RCTs) to examine apps that aimed to change health behaviors but did not exclude any studies based on a particular type of patient, health behavior, or app intervention. Thus, because their review only included RCTs and used broad search terms, many relevant mobile health apps were likely excluded.

Payne et al also conducted a systematic review to identify the current state of health behavioral intervention apps, which behavioral features they used, and their acceptability and effectiveness [17]. The majority of the studies included in the review examined apps for exercise and diet interventions; other interventions included mental health, diabetes management, and addiction. The authors concluded that the available evidence supported the acceptability and efficacy of most of the apps reviewed. However, they noted that most of the papers were pilot studies with small sample sizes, and further research was needed to provide more robust evidence of the utility of mobile health app interventions [17]. Their search strategy was focused on literature published until September 2014, and the state of mobile health app interventions is likely to have changed since then.

Therefore, there is a need for both an update and an expansion of these reviews, to reveal the current state of mobile health app technology, to consider the effectiveness, and to consider the behavioral change techniques that drive positive change. An overview of the different types of cutting-edge mobile app technologies and their uses will make it easier to identify the behavioral change techniques and strategies that may be most effective at engaging participants and improving health behaviors and outcomes.

This review will be focused on three main research questions. First, how effective are mobile health apps at improving and maintaining positive health behavior changes, and in which health behaviors are they the most effective? Second, what types of behavior change techniques are being used to support patient engagement with their health behaviors? Finally, what are patient perceptions of the feasibility, functionality, and overall user experience of the mobile health apps they use?

## Methods

### Overview

We will use the population, intervention, comparator, and outcome template and the Preferred Reporting Items for Systematic Reviews and Meta-Analyses Protocols (PRISMA-P) [18] to identify appropriate Medical Subject Headings (MeSH) for the literature search and structure the review. This systematic review will be composed of a literature search, article selection, data extraction, quality appraisal, data analysis, and data synthesis. This review was prospectively registered on PROSPERO (CRD42019155604).

### Eligibility Criteria

The following population, intervention, comparator, and outcome framework is based on the three research questions stated above.

- Population: The population will include people of any age (adults, adolescents, or children) who have interacted with the health care system or their health management using digital technologies or interventions. However, the review will focus on the general population, and specific subgroups such as pregnant women and patients with specific diseases (including HIV, posttraumatic stress disorder, alcoholism, and depression) will be excluded.
- Intervention: Mobile apps that are aimed at delivering interventions to improve and maintain positive health behaviors.
- Comparator: No specific comparator is required for studies to be included in this systematic review.
- Outcomes: The primary objective is to identify the types of mobile apps used in health care and behavioral change support and their effectiveness. This includes outcomes such as the extent and maintenance of behavior changes, adoption and adherence rates of the technology, patient-reported experience, feasibility and usability assessments, and coherence of the technology with behavioral change techniques.
- Studies: Due to an expectedly large volume of retrieved citations, the final set of included studies will be limited to randomized controlled trials.

### Search Strategy

We will search the following databases: Medline, Embase, CINAHL, and Web of Science. Key terms relating to digital health technologies were extracted from an initial review of the literature. Specific search terms, including health behaviors and health conditions, were identified in a preliminary scan of the literature and chosen in consultation with a medical librarian. Search terms will include MeSH terms and keywords related to mobile phones, mobile apps, health behaviors, and evaluation. Tablet devices capable of running mobile apps were not included in the search to narrow the scope, but studies that included the use of apps on mobile tablets were not excluded. The search terms that will be used in this review are grouped into four themes presented in Table 1. All terms in the MeSH and Keywords columns are included with the structure: Mobile (MeSH OR Keywords) AND Applications (MeSH OR Keywords) AND Health Behavior (MeSH OR Keywords) AND Evaluation (MeSH OR Keywords). Due to an unexpectedly large number of included studies, reference lists of included studies and grey literature will not be searched. Authors of conference and poster abstracts selected for inclusion will be contact to see if a full text is available to include.

**Table 1:** Search terms.

Category	MeSH^a^	Keywords (in title or abstract)
Mobile	Cell Phone OR Telemedicine	Smartphone OR “mobile phone” OR “mHealth” OR “mobile health”
Applications	Mobile Applications	App OR apps OR “mobile app*” OR “smartphone app*”
Health Behavior	Health Behavior OR Health Promotion OR Exercise OR Weight Loss OR Obesity (diet therapy, prevention & control, rehabilitation, therapy) OR Nutrition Therapy OR Diet OR Smoking Cessation OR Smoking Reduction OR Tobacco Use Cessation OR Alcohol Drinking (prevention & control, therapy) OR Mental Health OR Safe Sex OR Behavioral Medicine OR Chronic Disease	“Health behaviour” OR “health behavior” OR “behaviour change” OR “behavior change” OR (Exercise ADJ3 (increase or start or maintain*)) OR “physical activity” OR “Weight loss” OR “healthy weight” OR “five a day” OR “diet” OR “nutrition” OR ((Maintenance OR maintain* OR achiev* Or retain*) ADJ4 (weight goal OR “weight loss” OR “goal weight” OR BMI)) OR (Smoking ADJ4 (cessation OR stop* OR quit* OR reduc*)) OR (Alcohol ADJ4 (reduc* OR limit* OR decreas* OR “cutting down” OR “cut down” OR “cut back” OR less* OR curb* OR abstain OR “dry January”)) OR “Protection from sun” OR “sun protection” OR “sun safe*” OR ((Sex OR “sex* behaviour” OR “sex* behavior”) ADJ4 (safe* OR protect*)) OR ((Alzheimer* disease OR arthritis OR asthma OR cancer OR COPD OR Crohn* disease OR cystic fibrosis OR dementia OR diabetes OR epilepsy OR heart disease OR HIV OR AIDS OR mood disorders OR bipolar OR depression OR anxiety OR multiple sclerosis OR Parkinson* disease) ADJ4 (manag* OR “self help” OR “self manag*” OR coping OR cope)) OR “Health management”
Evaluation	Outcome Assessment (Health Care)	Feasibility OR usability OR “evaluat*” OR “outcome*” OR acceptability OR adherence OR “effectiv*” OR “adoption” OR “assess*”

^a^MeSH: Medical Subject Headings.

### Inclusion Criteria

We will include studies published between 2014 and 2019. Only the previous five years of research will be examined because technology has been evolving very rapidly, and this review is concerned with the current state of digital health technologies [19]. This period also provides an update to Payne et al’s systematic review that covered 2007-2014 [17]. Studies that evaluated at least one mobile app designed to monitor or improve patient health behaviors will be included. Any nationalities, geographic locations, or health behaviors will be included, provided that mobile app technologies are being used to help users improve, adopt, or maintain positive health behaviors and are the main focus of the study.

For the initial search, all study types will be included to ensure that no randomized controlled trials are missed. However, the studies included in the final review will be refined to include only RCTs.

### Exclusion Criteria

We will exclude studies that are not published in English, studies of mobile app interventions that are not focused on health behaviors, and studies of mobile apps designed for use by health care professionals. Studies will also be excluded if their focus is on behavior change theory without reference to mobile apps, or if they examine interventions that use mobile phones or wearable technology but do not involve apps (eg, interventions based solely on text messaging or emails). However, if the mobile app is the focus of the study, but data collection involves wearables, it will be included. Mobile apps will not be considered the main focus of the study when they are only a minor component of an intervention, and their independent effect on health behavior is not examined. Studies will also be excluded if they do not evaluate the mobile app(s) considered.

This review will focus on the general population, so specific subgroups such as pregnant women will be excluded. However, reviews will not be excluded if they focus on particular demographic subgroups (for instance, based on age or nationality).

### Screening and Article Selection

All the articles identified from the database searches will be stored in the citation management software Mendeley, which will be used to eliminate any duplicates. Two independent reviewers will screen the titles and abstracts of all the studies. Studies that fail to meet the eligibility criteria will be excluded, with any disagreements being discussed until there is consensus. The full text of the remaining studies will then be examined by one reviewer and validated by the other to determine final eligibility, with any disagreements being resolved by a third reviewer. A PRISMA flow diagram will be used to record the details of the screening and selection process so that the study can be reproduced.

### Data Extraction

One reviewer will examine the full text of all the papers included in the final selection to extract the predetermined outcomes, which will be validated by a second reviewer. Outcomes will be extraction into a preestablished, custom-built form. Disagreements will be resolved by discussion, and if consensus cannot be reached, a third reviewer will be consulted. The broad scope of the review means that there will likely be high heterogeneity in the data reported by various studies. However, an initial review of the literature has suggested various measures of effectiveness that are commonly used, which have been included in the data extraction table (see Textbox 1). Missing data will be considered in the risk of bias assessment, but due to time limitations, authors will not be contacted.

**Article information and data extracted.**

**General study information**

- Year of publication
- Countries of study
- Study setting (primary location of app use, if relevant)
- Analyzed sample size
- Sample demographics (including age, gender, target population)
- Intervention duration and follow-up periods

**Behavioral intervention**

- Target health behavior(s) and intervention focus
- Theory the intervention is based on
- Behavior change techniques [16,20]

**Mobile app technology**

- Area of health care used in
- Name of the app
- Developers
- Platform
- Components and design features (eg, provision of feedback, notifications, tracking)

**Evaluation**

- What outcomes were measured
- Participant health outcomes
- Behavior change outcomes
- Participant engagement/adherence rates
- Participant satisfaction
- Feasibility and usability
- Other key performance indicators reported

### Quality Appraisal and Risk of Bias Assessment

After the final selection of the studies, one reviewer will assess the risk of bias of all the papers included in the final selection. A quarter of the studies will be randomly selected for validation by a second reviewer. If there is disagreement in judgment, the reviewers will discuss before consulting a third reviewer. The Cochrane Collaboration Risk of Bias tool will be used to assess the randomized controlled trials included in the review and assign low, unclear, or high risk to the studies for each of the potential biases [21]. A table will be created summarizing the quality of all included studies.

### Data Analysis and Synthesis

It is unlikely that a meta-analysis will be feasible due to the anticipated variety of study aims, methods, and reported outcomes. Therefore, we will conduct a descriptive analysis to summarize the extracted data. Each health behavior and patient health outcome will be coded as having no, some, or significant evidence of effectiveness. Significant evidence will be coded only when the app performs significantly better than a comparator or control. The app will be considered to have some evidence of effectiveness if there is a significant difference over time but not between groups or a significant improvement in only a subgroup of the population. Health behaviors and patient health outcomes will be analyzed separately. Studies will be grouped by target health behavior (eg, smoking cessation) and analyzed together to describe the effectiveness of mobile health apps in general for those target behaviors, and in particular, for behavioral and health outcomes. The discussion will synthesize the data to describe the current state of mobile health apps, draw conclusions about their feasibility, usability, effectiveness, and use of behavioral change techniques, and identify limitations and directions for future research and development.

## Results

As of November 2019, the systematic review has been completed and is in peer review for publication.

## Discussion

A systematic and transparent review of the literature will provide a better understanding of current state-of-the-art mobile health apps, how they are being used, and to what effect. Strengths, limitations, and implications for the interaction of technology and behavioral health management will help inform and improve the development, acceptability, and effectiveness of future mobile health apps. Based on the data, this section will explore what conclusions can be drawn, what limitations exist in the systematic review, and what the important directions for future research are.

## Abbreviations

MeSH: Medical Subject Headings
mHealth: mobile health
PRISMA-P: Preferred Reporting Items for Systematic Reviews and Meta-Analyses Protocols
RCT: randomized controlled trial
